# Zika Virus–Associated Cognitive Impairment in Adolescent, 2016

**DOI:** 10.3201/eid2306.162029

**Published:** 2017-06

**Authors:** Jason Zucker, Natalie Neu, Claudia A. Chiriboga, Veronica J. Hinton, Marc Leonardo, Arif Sheikh, Kiran Thakur

**Affiliations:** Columbia University Medical Center, New York, New York, USA (J. Zucker, N. Neu, C.A. Chiriboga, V.J. Hinton, A. Sheikh, K. Thakur);; Child, Adolescent, and Adult Psychiatry private practice, New York (M. Leonardo)

**Keywords:** Zika virus, neuropsychiatric symptoms, cognitive impairment, flavivirus, adolescent, viruses, vector-borne infections

## Abstract

Incidence of neurologic manifestations associated with Zika virus infection has been increasing. In 2016, neuropsychological and cognitive changes developed in an adolescent after travel to a Zika virus–endemic area. Single-photon emission computed tomography and neuropsychological testing raised the possibility that Zika virus infection may lead to neuropsychiatric and cognitive symptoms.

Although the full clinical spectrum of complications associated with Zika virus disease remains unclear, evidence of an association between Zika virus infection and diseases of the nervous system is growing (*1*). Increased incidence of neurologic manifestations has been reported, including cerebral birth abnormalities (*2*), Guillain-Barré Syndrome (*3*), meningoencephalitis (*4*), and memory loss (*5*). We describe a case of probable central nervous system (CNS) infection with Zika virus associated with the onset of neuropsychological and cognitive changes in an adolescent. 

In summer 2016, an adolescent traveled to a Zika virus–endemic island in the southern Caribbean, where many insect bites occurred. Before the trip, the patient’s medical history included mild depression treated with a selective serotonin reuptake inhibitor. Assessment after return to the United States indicated that mental and physical health were the same as before travel. One week after return, the patient experienced sore throat, headache, diffuse scarlatiniform rash (Technical Appendix Figure 1), joint pain, confusion, and short-term memory loss; no fever or eye inflammation were noted.

Three days after symptom onset, the patient received care from a pediatrician; extensive laboratory testing revealed urine reverse transcription PCR (RT-PCR) results positive for Zika virus. Serologic results were positive for Epstein-Barr virus, consistent with the patient’s history of infection 15 months earlier (Technical Appendix Table 1). Five days after symptom onset, the sore throat, headache, rash and joint pain resolved; however, 8 days after symptom onset, the neuropsychiatric symptoms worsened and included excessive energy, decreased sleep, rapid and tangential speech, grandiose thinking, impulsivity, decreased inhibition, and behavioral regression suggestive of hypomania. The patient’s psychiatrist prescribed an antipsychotic medication. An ambulatory and 24-hour electroencephalogram and magnetic resonance imaging of the brain without contrast showed no abnormalities.

Symptoms failed to improve, and 13 days after symptom onset, the patient was hospitalized for further work-up. Fourteen days after symptom onset, magnetic resonance imaging of the brain with contrast showed no abnormalities. Serum was positive for dengue virus IgG and IgM (Technical Appendix Table 2). Repeat Zika virus testing was not performed because of the prior positivity of urine RT-PCR. Fifteen days after symptom onset, cerebrospinal fluid (CSF) obtained by lumbar puncture contained 8 leukocytes/μL, 1,000 erythrocytes/μL, 55 mg/dL glucose, and 30 mg/dL protein. CSF testing for Zika virus, performed at the Wadsworth Laboratory, New York City Department of Mental Health and Hygiene (New York, NY, USA), was positive for IgM but negative by RT-PCR.

After discharge, the patient’s symptoms of regression, disinhibition, and cognitive impairment persisted. Seven weeks after symptom onset, single-photon emission computed tomography revealed mildly heterogeneous cerebral cortical perfusion, with focal moderate-to-severe hypoperfusion in the inferior left frontal region (Technical Appendix Figure 2); neuropsychological testing demonstrated evidence of superior intellectual ability (Wechsler Intelligence Scale for Children–fourth edition, General Ability Index), probably reflecting function before illness (Technical Appendix Table 3). Processing speed was significantly slowed relative to other skills; the Processing Speed Index score was >2 SDs lower than the General Ability Index score. Performance on most memory tests and tests of executive function was within normal limits yet lower than expected given reported performance before illness. Immediate and delayed visual recall of the Rey complex figure was poor, reflecting primary difficulty encoding new visual information. On a standardized behavioral questionnaire, the patient self-reported psychiatric symptoms including anxiety, racing thoughts, and an inability to turn off thoughts. The patient had not experienced racing thoughts before the Zika virus infection, and the anxiety symptoms had worsened since the infection. In addition, the patient reported significant and functionally limiting fatigue. Nine weeks after onset, symptoms were better but not resolved; because of concerns that these symptoms were triggered by a postinfectious immune-mediated process, a trial of intravenous immunoglobulin was administered. Fifteen weeks after symptom onset, the patient’s symptoms were better but not fully resolved.

Although we cannot entirely rule out Epstein-Barr virus as a possible trigger, the length of time between previous infection and onset of neuropsychological symptoms would be unusual. In addition, although it is impossible to exclude contributions of coinfection from other mosquitoborne viruses (e.g., dengue and chikungunya), given the Zika virus positivity on RT-PCR, the patient’s condition met criteria for definitive Zika virus infection and the CSF IgM titer was consistent with CNS involvement of Zika virus. The changes on single-photon emission computed tomographs and neuropsychological test scores raise the possibility that Zika virus infection may trigger neuropsychiatric and cognitive symptoms. Although we cannot prove that the patient’s symptoms were related to Zika virus, clinicians should be aware of this potential association and the value of closely monitoring patients with Zika virus infection.

Technical Appendix Outpatient and inpatient laboratory results, neuropsychological test results, photograph of rash, and single-photon emission computed tomography and magnetic resonance images for an adolescent returning from a Zika virus–endemic area, 2016.
