# Supplementary material for: Zika Virus–Associated Cognitive Impairment in Adolescent, 2016
Source: Emerg Infect Dis. 2017 Jun;23(6):1047–8. doi: 10.3201/eid2306.162029 (PMC5443451; doi:10.3201/eid2306.162029)
Supplement: Technical Appendix — Outpatient and inpatient laboratory results, neuropsychological test results, photograph of rash, and single-photon emission computed tomography and magnetic resonance images for an adolescent returning from a Zika virus–endemic area, 2016. [file 16-2029-Techapp-s1.pdf]

Article DOI: https://dx.doi.org/10.3201/eid2306.162029

Zika Virus–Associated Cognitive Impairment in Adolescent, 2016

Technical Appendix

Technical Appendix Table 1. Outpatient laboratory evaluation of acute illness and mental status changes in an adolescent returning from a Zika endemic territory

Date	Test (units)	Result	Reference
8/6/16*	EBV EBNA (U/ml)	75.4 (H)	0–21.9
	EBV VCA IgG (U/ml)	100 (H)	0–21.9
	EBV VCA IgM (U/ml)	<10	0–43.9
	EBV IgG (U/ml)	9.3	0–10.9
	Mycoplasma IgG (U/L)	.02	<0.09
	Mycoplasma IgM (U/L)	.21 U/L	<0.76
	Parvovirus IgG (IV)	5.73 (H)	<0.89
	Parvovirus IgM (IV)	.24	<0.89
	Antinuclear antibody	Negative	Negative
	Antistreptolysin O Titer (IU/ml)	63	0–330
	DNase B antibody (U/ml)	169	0–310
	Sedimentation rate (mm/hr)	8	0–24
	Thyroid stimulating hormone (uIH/mL)	1.2	.4 – 4.2
	Vitamin B12 (pg/ml)	406	211–911
	Folate (ng/mL)	10.17	4–1000
	Vitamin D 25 Hydroxy (NG/ml)	42.5	30–100
8/6/16†	Lyme Western Blot IgM	Negative	Neg
	Lyme Western Blot IgG	Negative	Neg
	Serum N methyl D Aspartate Receptor Antibody	<1:10	<1:10
8/6/16‡	Zika Serum RT-PCR	Negative	Negative
	Zika Serology (IgM)	Negative	Negative
	Zika Urine RT-PCR	Positive	Negative

*Labs performed at Mount Sinai Beth Israel Laboratory.
†Labs performed at an outside hospital laboratory.
‡Labs performed at New York Department of Health.

Technical Appendix Table 2. Inpatient laboratory evaluation of acute illness and mental status changes in an adolescent returning from a Zika endemic territory

Date	Test	Result	Reference
Serum 8/16/16*	Urine toxicology	Negative	Negative
	C-Reactive Protein (mg/L)	<0.15	0–10
	Sedimentation rate (mm/hr)	10	0–20
	Dengue Fever Virus Antibody IgM (IV)	2.15 (H)	<1.64
	Dengue Fever Virus Antibody IgG (IV)	14 (H)	<1.64
	Total IgG (mg/dL)	941	714 - 1711
CSF 8/18/16*	CSF Cell Count (cells/microliter)	8 WBCs	0 - 5
		1000 RBCs	0
	CSF Glucose (mg/dL)	55 (L)	60 - 80
	CSF IgG and Total Protein Ratio	6.7	6–13
	BioFire® Meningitis/Encephalitis RT- PCR Panel	Negative	Negative
	Oligoclonal Bands	Negative	Negative
8/18/16*	CSF N methyl D Aspartate Receptor Antibody, IgG	Negative	Negative
8/18/16†	CSF Zika RT-PCR	Negative	Negative
	CSF Zika IgM	Positive	Negative

*Labs performed at New York Presbyterian Columbia.
†Labs performed at New York Department of Health.

Technical Appendix Table 3. Neuropsychological test findings

Intellectual Function	Score	Percentile
Wechsler Intelligence Scale for Children - 5	Standard Score	Percentile
Verbal Comprehension	118	88
Visual Spatial	117	87
Fluid Reasoning	118	88
Working Memory	115	84
Processing Speed	83	13
General Ability Index	121	92
Memory and Learning		
California Verbal Learning Test for Children	T/z-score	Percentile
Trials 1–5	45	34
Long Delay Free Recall	0	50
NEPSY - 2	Scaled Score	Percentile
Memory for Designs	12	75
Memory for Designs-Delayed	13	84
Rey Complex Figure Test	T-score	Percentile
Immediate Recall	29	2
Delayed Recall	24	1
Recognition	27	1
Wide Range Assessment of Memory and Learning 2	Scaled Score	Percentile
Story Memory	11	63
Story Memory Delay Recall	12	75
Story Memory Recognition	14	91
Executive Functions		
Delis-Kaplan Executive Function System	Scaled Score	Percentile
Verbal Fluency		
Letter	14	91
Category	12	75
Switching	13	84
Trail Making		
Visual Scanning	7	16
Number Sequencing	10	50
Letter Sequencing	12	75
Number-Letter Switching	8	25
Motor Speed	10	50
Color Word Interference		
Color Naming	9	37
Word Reading	11	63
Inhibition	8	25
Inhibition - Total Errors	8	25
Inhibition/Switching	9	37
Inhibition/Switching - Total Errors	10	50
Tower		
Total Achievement Score	13	84
Mean First Move Time	13	84
Time-Per-Move Ratio	11	63
Move Accuracy Ratio	8	25

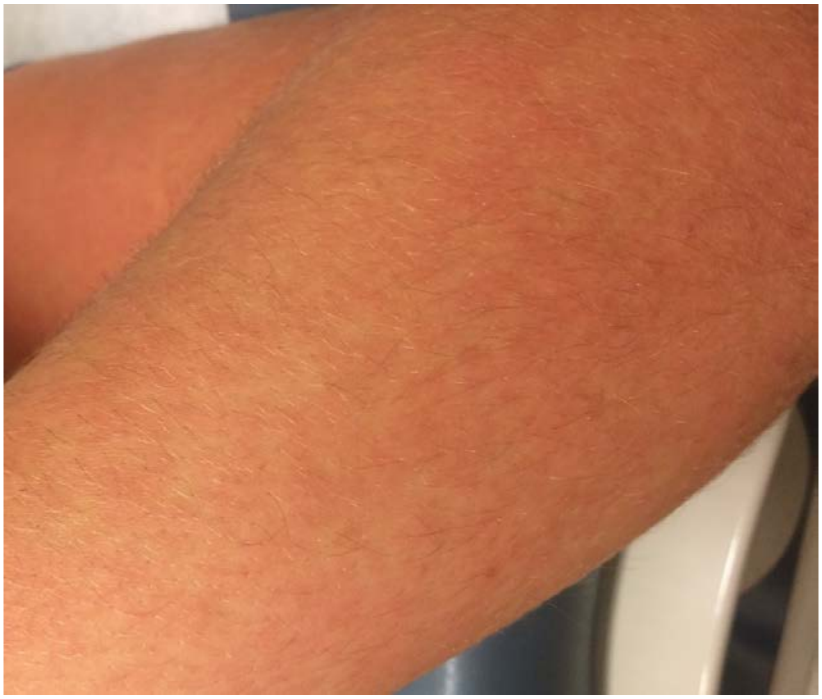

Technical Appendix Figure 1. Scarlitiform rash in a patient with urine positive for Zika virus on reverse transcription PCR. Picture taken 4 days after the onset of symptoms.

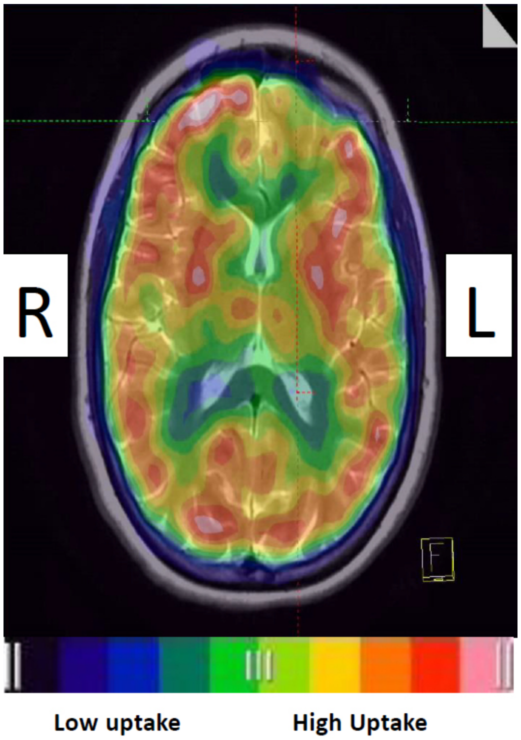

Technical Appendix Figure 2. This ^{99m}Tc-ethyl cysteinate dimer (ECD) fusion of brain SPECT and MRI shows the localization of focal left inferofrontal lobe moderate-severely decreased hypoperfusion (crosshairs), along with a non-specific heterogeneous global uptake pattern.
